# How familial Mediterranean fever affects the characteristics of immunoglobulin a vasculitis in pediatric patients at the time of diagnosis?

**DOI:** 10.1007/s00431-025-06091-y

**Published:** 2025-04-07

**Authors:** Mehveş Işıklar Ekici, Elif Çelikel, Zahide Ekici Tekin, Vildan Güngörer, Cüneyt Karagöl, Melike Mehveş Kaplan, Nimet Öner, Merve Cansu Polat, Didem Öztürk, Emine Özçelik, Yasemin Uğur Es, Sultan Nilay Yoğun, Banu Çelikel Acar

**Affiliations:** Division of Pediatric Rheumatology, Department of Pediatrics, University of Health Sciences, Ankara Bilkent City Hospital, Bilkent, Ankara, 06800 Turkey

**Keywords:** Familial Mediterranean fever, Vasculitis, Immunoglobulin A vasculitis, Pediatrics

## Abstract

Familial Mediterranean fever (FMF) is the most common autoinflammatory disease of childhood and is frequently associated with systemic inflammatory diseases such as vasculitis. The aim of this study is to evaluate the impact of FMF on clinical and laboratory findings and disease activity of immunoglobulin A vasculitis (IgAV) at presentation. Patients with IgAV who were followed up for more than 3 months were included in the study. Patients were divided into two subgroups regarding presence of FMF. Demographic characteristics, clinical findings in the first 3 months, laboratory findings at the time of diagnosis, vasculitis activity scores (PVAS), and treatments administered were recorded. A total of 662 patients with IgAV were included in the study. FMF was diagnosed in 49 (7.4%) patients with IgAV. Patients with FMF had more gastrointestinal tract and renal involvement, higher PVAS score, higher C-reactive protein levels, and higher need for steroids, cyclophosphamide, and intravenous immunoglobulin (p = 0.01, p = 0.03, p < 0.001, p < 0.001, p = 0.04, p < 0.001, p = 0.01, respectively). When patients were analyzed according to MEFV mutations, the effect of FMF on vasculitis was more prominent especially in patients carrying homozygous and compound heterozygous mutations in exon 10.

*Conclusion*: FMF may lead to more severe clinical and laboratory findings and disease activity at the time of IgAV diagnosis. The clinician should be aware that the course of IgAV may be affected by the presence of FMF.

*Trial registration*: E2-24–6125, 21.01.2024, retrospectively registered.

**Table:** 

**What is Known:** • *In patients with familial Mediterranean fever (FMF), some vasculitides are more common and may affect the course of vasculitis.* **What is New:** • *At the time of immunoglobulin A vasculitis diagnosis, patients with FMF may have more system involvement, higher vasculitis activation scores, and acute phase reactants and require more intensive treatment than those without FMF.*

**Supplementary Information:**

The online version contains supplementary material available at 10.1007/s00431-025-06091-y.

## Introduction

Familial Mediterranean fever (FMF) is the most common autoinflammatory disease of childhood characterized by self-limiting fever, serositis, and arthritis attacks. The disease is caused by a mutation in the Mediterranean fever gene (*MEFV*) encoding the pyrin protein. The mutated pyrin leads to an increased inflammatory response as a result of release of IL-1β [1, 2].

Uncontrolled IL-1 release in FMF is considered to play a role by lowering the threshold of inflammation required for the development of some systemic diseases [2]. Over the years, FMF has been shown to contribute to the development or alter the course of many systemic diseases such as inflammatory bowel disease (IBD), ankylosing spondylitis (AS), and vasculitis [3, 4]. It was reported that the presence of *MEFV* mutation in immunoglobulin A vasculitis (IgAV) patients affected the clinical course of the disease; gastrointestinal tract and renal involvement was more frequent in these patients and acute phase reactants may be higher at presentation [5]. On the other hand, it has also been shown that the presence of a mutation in the *MEFV* gene is not associated with laboratory or clinical manifestations of IgAV [6].

Understanding the interaction between FMF and IgAV is crucial as it can significantly influence disease manifestations, management strategies, and prognosis. The aim of this study is to evaluate the impact of FMF on clinical and laboratory findings and disease activity of IgAV at presentation. In addition, the effect of *MEFV* mutations on the characteristics of IgAV at onset is investigated in detail.

## Material method

This retrospective medical record review study was conducted on patients diagnosed with IgAV who were followed up for at least 3 months in the Pediatric Rheumatology Department of our hospital between 2013 and 2024. Patients fulfilling the 2008 Ankara PreS/EULAR/PRINTO diagnostic criteria for IgAV were included [7]. Patients were subgrouped according to the presence or absence of FMF. Patients with missing data and those with a follow-up period of less than 3 months were excluded (Flowchart 1).

Patients who met Eurofever classification criteria for FMF were enrolled in the study as patients with FMF [8]. According to Eurofever classification criteria, FMF was diagnosed in two different ways according to the presence or absence of a confirmed *MEFV* gene mutation [8]. Patients who did not carry the *MEFV* mutation and had a homozygous or heterozygous mutation of unknown significance such as E148Q but showed clinical features of FMF were included in the study as clinically diagnosed patients.

### Data collection

Patients’ demographic characteristics, clinical findings, whether or not FMF was diagnosed, laboratory findings at the time of diagnosis, *MEFV* results, Pediatric Vasculitis Activity Score (PVAS), and treatments given were recorded from electronic medical records. The onset of IgAV is usually acute and the main clinical manifestations develop within a few days to a few weeks. However, the initial 3 months are critical for system involvement, especially renal involvement [9]. Therefore, clinical and systemic involvements of all patients were recorded in the first 3 months after the presentation of IgAV.

*MEFV* gene analysis was performed with the Sanger sequencing method by the Genetics Department of our hospital. Exons 2, 3, 5, and 10 of the *MEFV* gene were analyzed.

### Definition

Vasculitis severity was assessed using PVAS, a scoring system based on a total of 64 parameters in nine main categories used to measure disease activation and treatment response. Increased PVAS indicates more active disease [10].

### Study approval

The study was conducted in accordance with the principles of the Declaration of Helsinki. The study was approved by the institutional ethics committee (ethics approval number: E2-24–6125).

### Statistical analyses

**Fig. 1:**
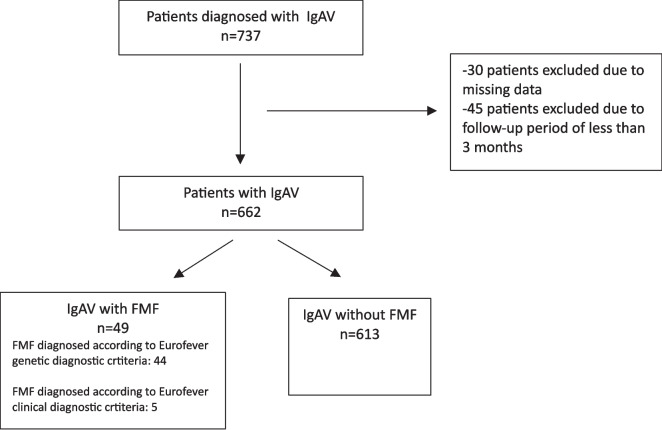
Patient selection process

Mean ± SD values were used for normally distributed variables, while median (IQR) values were used for variables that did not show normal distribution. The Mann–Whitney *U* test was used for two group comparisons of continuous data. Chi-square and Fisher’s exact tests were used for group comparisons of nominal variables. Version22 (SPSS Inc., Chicago, IL) program was used in the evaluations and *p* < 0.05 was accepted as the statistical significance limit (Fig. 1).

## Results

The data of 737 patients who were followed up with the diagnosis of IgAV during the study period were reviewed. Forty-five patients with a follow-up period of less than 3 months and 30 patients with missing data were excluded from the study. The remaining 662 patients with IgAV were included in the study.

Three hundred and eleven (47%) of IgAV patients were female. The median age at diagnosis was 7.2 (4.1–17) years. The median follow-up period was 1.2 (0.8–15) years. All IgAV patients had skin involvement. There were gastrointestinal tract involvement in 245 (37%) patients, arthralgia/arthritis in 200 (30.2%), renal involvement in 54 (8.1%), and scrotal involvement in 39 (5.9%). The median PVAS was 2 (1–5).

The patients were subgrouped according to the presence of FMF. Of the 662 IgAV patients, 49 (7.4%) had FMF. The *MEFV* results of the patients are given in Table 1.

**Table 1:** *MEFV* mutations in IgAV patients with FMF

	IgAV patients with FMF *n* = 49 (7.4%)
***M694V homozygous*** ***Exon 10 compound heterozygous*** M794V/M680I M694V/V726A V726A/V726A ***Exon 10 heterozygous*** M694V/- V726A/- M680I/- ***Exon 2 homozygous*** E148Q/E148Q ***Exon 2 heterozygous*** E148Q/- ***No mutation***	14 10 5 2 3 20 12 5 3 2 2 1 1 2

*IgAV* immunoglobulin A vasculitis,* FMF* familial Mediterranean fever,* MEFV* Mediterranean fever gene

Twelve of 49 (24.5%) IgAV patients with FMF were followed up with a diagnosis of FMF prior to vasculitis. *MEFV* gene analysis was performed in 107 IgAV patients during vasculitis follow-up. The reason for *MEFV* mutation screening was widespread rash in 17 (15.9%), severe systemic involvement in 84 (78.5%), and recurrent leukocytoclastic vasculitis in 6 (5.6%). As a result, 35 (32.7%) patients who met Eurofever genetic classification criteria and 2 patients who met Eurofever clinical classification criteria were diagnosed with FMF at the time of IgAV diagnosis. Of the 107 patients who were analyzed for *MEFV* gene, 70 patients were not considered as FMF because 43 patients had no mutation and 27 patients had heterozygous mutation but no clinical symptoms.

Demographic characteristics, clinical and laboratory findings, PVAS, and treatments of IgAV patients with and without FMF are given in Table 2. The rate of gastrointestinal tract and renal involvement, median PVAS, median C-reactive protein (CRP), and need for steroids, cyclophosphamide, intravenous immunoglobulin (IVIG), and plasma exchange therapy were significantly higher in IgAV patients with FMF (*p* = 0.01, *p* = 0.03, *p* < 0.001, *p* < 0.001, *p* = 0.04, *p* < 0.001, *p* = 0.01, *p* < 0.001, respectively). When patients who were carriers of the *MEFV* mutation but did not show clinical features of FMF were compared with patients without the mutation, no significant difference was found in terms of laboratory findings, clinical features, and vasculitis activity scores (Table 3).

**Table 2:** Comparison of demographic characteristics, clinical and laboratory findings, PVAS, and treatment of IgAV patients with and without FMF in the first 3 months after presentation of IgAV

	**Total IgAV patients** **n=662**	IgAV patients with FMF *n*=49	IgAV patients without FMF *n*=613	*p* value
Median age at diagnosis of IgAV, years	7.2 (4.1–17)	7.3 (4.3–15)	7.2 (4.1–17)	0.98
Sex, n (%)				
Female	311 (47)	21 (3.2)	290 (44.8)	0.36
Male	351 (53)	28 (4.2)	323 (48.8)	0.36
Gastrointestinal tract involvement, n (%)	245 (37)	27 (54)	218 (35.6)	0.01
Arthritis, n (%)	200 (30.2)	18 (36.7)	182 (29.7)	0.63
Renal involvement, n (%)	54 (8.1)	8 (16.3)	46 (7.5)	0.03
Scrotal involvement, n (%)	39 (5.9)	3 (6.1)	36 (5.7)	0.96
Laboratory findings				
Median WBC, x10^3^ /mm^3^	10.4 (7–14.4)	10.7 (9–14.1)	10.1 (7–14.4)	0.29
Median Hb, g/dL	12.5 (11.8–14.7)	12.4 (11.8–13.5)	12.7 (11.9–14.7)	0.16
Median PLT, x10^3^ /mm^3^	388 (328–895)	365 (340–802)	410 (328–895)	0.93
Median ESR, mm/h	25 (2–86)	25 (3–86)	22 (2–60)	0.95
Median CRP, mg/L	1.5 (0.5–134)	17.9 (5–134)	1.2 (0.5–80)	<0.001
PVAS, median	2 (1–5)	3 (1–5)	2 (1–5)	<0.001
Treatment, n (%)				
Steroid	319 (48.1)	32 (65.3)	287 (46.8)	0.04
Pulse methylprednisolone	58 (8.8)	15 (30.6)	43 (7)	<0.001
Cyclophosphamide	22 (3.3)	9 (18.4)	13 (2.1)	<0.001
İntravenous immunoglobulin	10 (1.5)	8 (16.3)	2 (0.3)	0.01
Plasma exchange therapy	2 (0.3)	2 (4)	-	-

*IgAV* immunoglobulin A vasculitis, *FMF* familial Mediterranean fever, *WBC* white blood cell count, *Hb* hemoglobin, *PLT* platelets, *ESR* erythrocyte sedimentation rate, *CRP* C-reactive protein, *PVAS* Pediatric Vasculitis Activity Score

**Table 3:** Comparison of IgAV patients who are carriers of the MEFV mutation but do not show clinical features of FMF and patients without the mutation

	IgAV patients without *MEFV* mutations without FMF *n*=635	IgAV patients with *MEFV* mutations without FMF^*^ n=27	*p* value
Gastrointestinal tract involvement, n (%)	210 (33)	8 (29.6)	0.08
Arthritis, n (%)	175 (27.6)	7 (25.9)	0.09
Renal involvement, n (%)	44 (6.9)	2 (7.4)	0.45
Scrotal involvement, n (%)	34 (5.3)	2 (7.4)	0.06
Median CRP, mg/L	1.2 (2.1–134)	2.5 (0.5–115)	0.48
PVAS, median	2 (1–5)	2 (1–5)	0.56
Treatment, n (%)			
Steroid	275 (43.3)	12 (44.4)	0.08
Pulse methylprednisolone	41 (6.1)	2 (7.4)	0.12
Cyclophosphamide	12 (1.9)	1 (3.7)	0.53
İntravenous immunoglobulinPlasma	2 (0.3)	-	1
exchange therapy	-	-	-

*IgAV* immunoglobulin A vasculitis, *FMF* familial Mediterranean fever, *CRP* C-reactive protein, *PVAS* Pediatric Vasculitis Activity Score

^a^MEFV gene mutations including E148Q, M694V, M680I, V726A, R761H, and K695R heterozygous mutations

According to *MEFV* gene mutations, patients with FMF were subdivided into those with homozygous/compound heterozygous mutation in exon 10 (*n* = 24), those with heterozygous mutation in exon 10 (*n* = 20), and those with mutation in exon 2 or without mutation but clinically diagnosed with FMF (*n* = 5). The rate of gastrointestinal tract involvement, median CRP and PVAS, and need for pulse methylprednisolone, cyclophosphamide, IVIG, and plasma exchange therapy were significantly higher in patients with homozygous/compound heterozygous mutation in exon 10 compared to IgAV patients without FMF (*p* = 0.04, *p* < 0.001, *p* = 0.01, *p* < 0.001, *p* = 0.01, *p* < 0.001, *p* < 0.001, respectively). When patients with heterozygous mutations in exon 10 were compared with those without FMF, renal and gastrointestinal tract involvement rates, median CRP and PVAS, and the need for steroids, pulse methylprednisolone, and cyclophosphamide were significantly higher (*p* = 0.03, *p* = 0.04, *p* = 0.04, *p* = 0.01, *p* = 0.01, *p* < 0.001, *p* = 0.02, respectively). In the group including patients with exon 2 mutation and patients without *MEFV* mutation, the rate of joint involvement and median CRP values were significantly higher (*p* = 0.02, *p* = 0.03, respectively), while no significant difference was found in terms of other organ involvement rates, median PVAS, and treatment options. The comparison of patients with FMF between subgroups according to *MEFV* mutations is shown in Tables 4 and  5.

**Table 4:** Comparison of IgAV patients with and without FMF according to MEFV genotype

	IgAV patients without FMF *n* = 613	IgAV patients with FMF *n* = 49					
		Exon 10 homozygous/compound heterozygous *n* = 24	*p* value	Exon 10 heterozygous *n* = 20	*p* value	Other* n = 5	*p* value
Gastrointestinal tract involvement, *n* (%)	218 (35.6)	13 (54.2)	0.04	13 (65)	0.04	1 (20)	0.46
Scrotal involvement, *n* (%)	36 (5.7)	2 (8.3)	0.62	2 (10)	0.86	-	0.57
Gastrointestinal tract involvement, *n *(%)	218 (35.6)	13 (54.2)	0.04	13 (65)	0.04	1 (20)	0.46
Scrotal involvement,* n* (%)	36 (5.7)	2 (8.3)	0.62	2 (10)	0.86	-	0.57
Renal involvement, *n* (%)	46 (7.5)	2 (8.3)	0.91	5 (25)	0.03	-	0.51
Arthritis,* n* (%)	182 (29.7)	10 (41.7)	0.22	4 (20)	0.33	4 (80)	0.02
Median CRP, mg/L	1.2 (0.5–80)	24 (7–103.8)	<0.001	12 (5–134)	0.04	10.9 (5–63)	0.03
Median PVAS	2 (1–5)	3 (1–5)	0.01	3 (1–4)	0.01	2 (1–4)	0.96
Treatment, *n *(%)							
Steroid	287 (46.8)	14 (58.3)	0.27	15 (75)	0.01	3 (60)	0.56
Pulse methylprednisolone	43 (7)	8 (33.3)	<0.001	7 (35)	<0.001	-	0.54
Cyclophosphamide	13 (2.1)	7 (29.2)	0.01	2 (10)	0.02	-	0.74
İntravenous immunoglobulin	2 (0.3)	8 (33.3)	<0.001	-	1	-	1
Plasma exchange therapy	-	2 (8.3)	<0.001	-	-	-	-

*IgAV* immunoglobulin A vasculitis, *FMF* familial Mediterranean fever, *CRP* C-reactive protein, *PVAS* Pediatric Vasculitis Activity Score

*Patients who have no mutation in MEFV gene but clinically diagnosed with FMF and patients with exon 2 mutations

**Table 5:** Effects of *MEFV* mutations on clinical and laboratory findings, PVAS, and treatment of IgAV patients with FMF

	Patients with FMF			*p*^b^ value
	Exon 10 homozygous/compound heterozygous *n* = 24	Exon 10 heterozygous *n* = 20	Other^a^ *n* = 5	
Gastrointestinal tract involvement, *n* (%)	13 (54.2)	13 (65)	1 (20)	0.01
Scrotal involvement, *n* (%)	2 (8.3)	2 (10)	-	0.89
Renal involvement, *n* (%)	2 (8.3)	5 (25)	-	0.04
Gastrointestinal tract involvement,* n* (%)	13 (54.2)	13 (65)	1 (20)	0.01
Scrotal involvement, *n* (%)	2 (8.3)	2 (10)	-	0.89
Renal involvement,* n *(%)	2 (8.3)	5 (25)	-	0.04
Arthritis,* n* (%)	10 (41.7)	4 (20)	4 (80)	0.02
Median CRP, mg/L	24 (7–103.8)	12 (5–134)	10.9 (5–63)	<0.001
Median PVAS	3 (1–5)	3 (1–4)	2 (1–4)	<0.001
Treatment				
Steroid	14 (58.3)	15 (75)	3 (60)	0.08
Pulse methylprednisolone	8 (33.3)	7 (35)	-	0.01
Cyclophosphamide	7 (29.2)	2 (10)	-	<0.001
İntravenous immunoglobulin	8 (33.3)	-	-	<0.001
Plasma exchange therapy	2 (8.3)	-	-	<0.001

*IgAV* immunoglobulin A vasculitis, *FMF* familial Mediterranean fever, *CRP* C-reactive protein, *PVAS* Pediatric Vasculitis Activity Score

^a^Patients who have no mutation in MEFV gene but clinically diagnosed with FMF and patients with exon 2 mutations

^b^Comparison of the IgAV patients according to MEFV gene genotypes

## Discussion

Familial Mediterranean fever is the most common autoinflammatory disease of childhood. It is known that FMF can be accompanied with some systemic inflammatory diseases such as vasculitis and it might affect the disease course. In this study, it was shown that IgAV patients with FMF had more gastrointestinal tract and renal involvement, higher vasculitis activity score, and the need for intensive treatment compared to patients without FMF. In addition, IgAV patients with homozygous/compound heterozygous or heterozygous mutations in exon 10 had significantly higher rates of gastrointestinal tract involvement, and CRP and PVAS values compared to those without FMF, whereas similar results for gastrointestinal tract involvement and PVAS were not observed in FMF patients without exon 10 mutations.

Familial Mediterranean fever may predispose to the development and affect the course of inflammatory diseases such as vasculitis, IBD, AS, and multiple sclerosis [3, 4, 11]. Although the etiopathogenesis of FMF-associated IgAV is unclear, genetic predisposition and various environmental factors are considered to play a role [12]. Mutations in the *MEFV* gene in FMF activate innate immunity through the formation of the pyrin inflammasome and leading to an increase in proinflammatory cytokines such as IL-1 β, IL-6, and IL-18 [13]. Interestingly, these cytokines have also been reported to be elevated in IgAV patients [14–16]. As a result, these two different diseases may share a common immunologic mechanism in pathogenesis. *MEFV* gene mutations can drive adaptive and immune responses through innate immune activation and may have an impact on the course of IgA vasculitis [17, 18]. In our study, it was found that gastrointestinal tract and renal involvement was significantly more frequent and PVAS and CRP levels were higher in IgAV patients with FMF. Our results indicate that FMF may cause a more severe course of IgAV. Altug et al. [19] found that *MEFV* mutation was detected in 26% of 68 patients with IgAV and gastrointestinal system involvement, arthritis, and subcutaneous edema were more common in these patients. In another study, *MEFV* mutation was found in 34% of 80 IgAV patients and those with the mutation were younger at the time of diagnosis of vasculitis and had more arthritis and subcutaneous edema than those without the mutation. Moreover, those with *MEFV* mutations were reported to have higher acute phase responses than those without [20]. Cakici et al. [5] reported that *MEFV* mutations in IgAV patients led to more arthritis, abdominal pain, scrotal involvement, and relapses and higher acute phase reactant levels, but did not affect renal involvement. On the other hand, there are also studies that found that whether or not *MEFV* mutations are carried in IgAV patients has no effect on demographic characteristics, and clinical and laboratory findings [6, 21]. Even considering the results of a few conflicting studies, IgAV patients with FMF tend to develop more system involvement and have a higher acute phase response and disease activity score. Awareness of the effect of FMF on IgAV may help the clinician to be alert to the unfavorable effects of the association between IgAV and FMF. Especially in patients with FMF prior to the diagnosis of IgAV, it may be tempting to predict the course from the first days of vasculitis diagnosis.

Phenotype-genotype correlation has been clearly established in FMF [22]. Patients carrying exon 10 mutations such as M694V and M680I are known to have a severe FMF phenotype, but exon 2 mutations such as E148Q are associated with a milder disease course [23]. Moreover, patients with homozygous and compound heterozygous mutations have a more severe phenotype than those with heterozygous mutations [24]. Although the genetic code of FMF is gradually being decoded and its association with FMF clinic has been revealed, the effect of these mutations on other systemic diseases is not clear. It is very intriguing whether the vasculitis phenotype and *MEFV* genotype correlation can be established in diseases such as vasculitis, which are predicted to affect the course of FMF. Results from a recent large cohort of 1120 patients with IgAV revealed that patients with a mutation in exon 10 of the *MEFV* gene were more likely to have abdominal pain, joint involvement, scrotal involvement, and higher acute phase reactants. Exon 2 mutations were reported to have no clinical or laboratory impact on the course of vasculitis (6). In the present study, patients with homozygous/compound heterozygous or heterozygous mutations in exon 10 had significantly higher median CRP and PVAS, gastrointestinal tract involvement rate, and need for intensive treatment compared to IgAV patients without FMF. In IgAV patients with FMF carrying exon 2 mutation or without *MEFV* mutation, joint involvement was more frequent and median CRP values were significantly higher compared to IgAV patients without FMF. That is, IgAV patients with FMF who did not carry the exon 10 mutation did not have more major organ involvement or higher PVAS. In countries where FMF is common, gene analysis of *MEFV* mutation carriers should be evaluated delicately. In our study, it was found that patients who carried mutations in the *MEFV* gene but did not show FMF clinical features did not differ from those without mutations in terms of laboratory findings, clinical features, and vasculitis activity in the first 3 months of vasculitis. This indicates that the presence of FMF rather than carrying the *MEFV* gene mutation may have an effect on IgAV.

This study has some limitations. Its retrospective nature and being conducted in a single center are its main limitations. However, demonstration of the effect of FMF on the course of vasculitis with our large cohort and quantitative evaluation of disease severity with PVAS are the strengths of our study.

## Conclusion

Familial Mediterranean fever may lead to more severe clinical and laboratory findings in patients with IgAV at diagnosis. These patients may have more gastrointestinal tract and renal involvement and higher vasculitis activity scores compared to those without FMF. The impact of FMF on IgAV is more prominent especially in patients carrying mutations in exon 10. Genetic analysis should be performed if FMF-related symptoms are suspected. The clinician should keep in mind that the presence of FMF may affect vasculitis activity and the presentation of IgAV at the time of diagnosis.

## Supplementary Information

Below is the link to the electronic supplementary material.

ESM 1 (JPEG 580 KB)

## Data Availability

No datasets were generated or analysed during the current study.

## Abbreviations

AS: Ankylosing spondylitis
CRP: C-reactive protein
FMF: Familial Mediterranean fever
IBD: Inflammatory bowel disease
IgAV: Immunoglobulin A vasculitis
IVIG: Intravenous immunoglobulin
MEFV: Mediterranean fever gene
PVAS: Pediatric Vasculitis Activity Score
