# Supplementary figures and images for: How familial Mediterranean fever affects the characteristics of immunoglobulin a vasculitis in pediatric patients at the time of diagnosis?
Source: Eur J Pediatr. 2025 Apr 7;184(5):282. doi: 10.1007/s00431-025-06091-y (PMC11976343; doi:10.1007/s00431-025-06091-y)

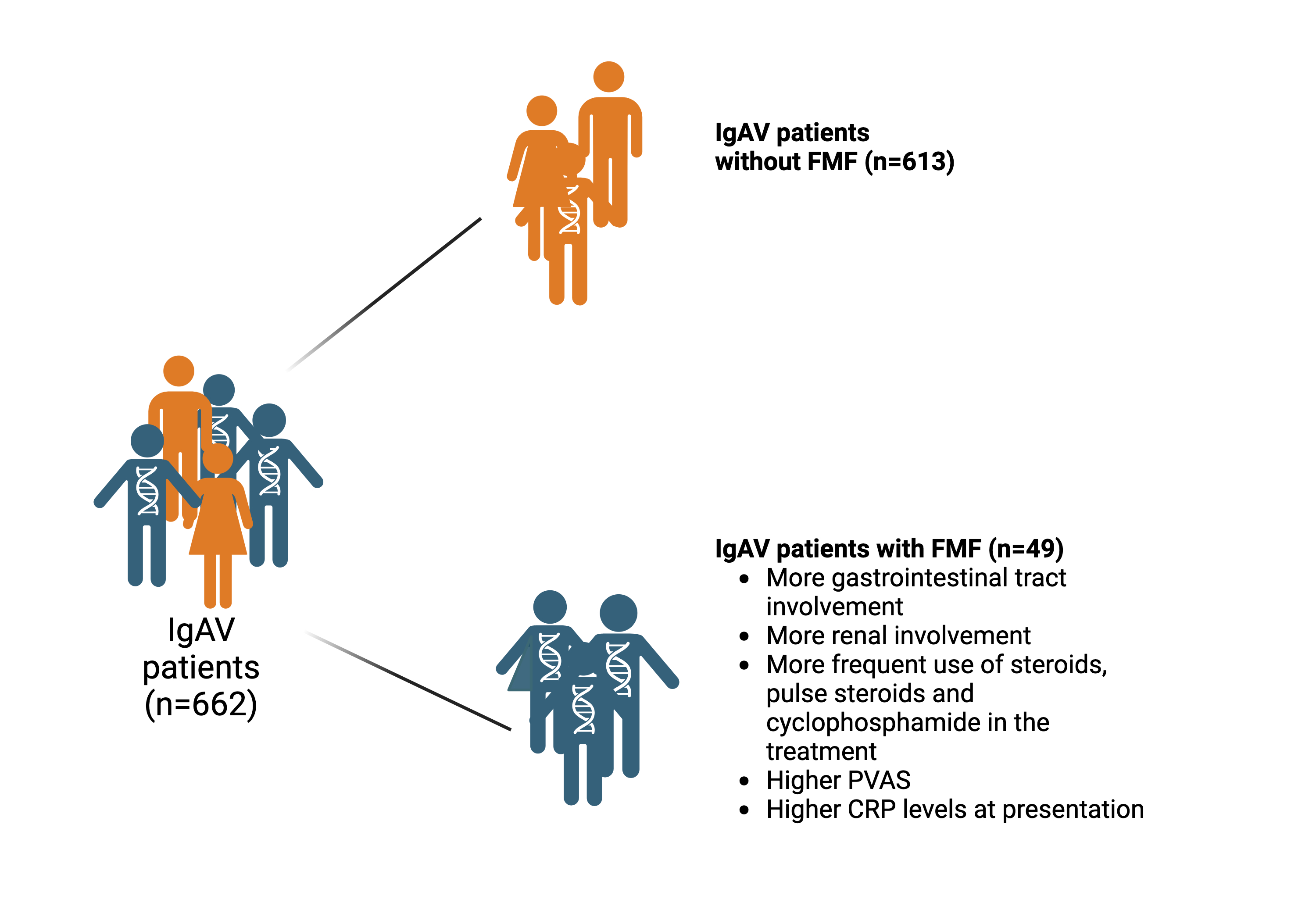

Supplement: Supplementary file 1 — (JPEG 580 KB) [file 431_2025_6091_MOESM1_ESM.jpeg]
